# Serial assessment of endothelial function 1, 6, and 12 months after ST-elevation myocardial infarction

**DOI:** 10.1007/s00380-018-1145-1

**Published:** 2018-03-14

**Authors:** Jasveen J. Kandhai-Ragunath, Carine J. M. Doggen, Liefke C. van der Heijden, Marlies M. Kok, Paolo Zocca, Bjorn de Wagenaar, Cees Doelman, Harald T. Jørstad, Ron J. G. Peters, Clemens von Birgelen

**Affiliations:** 1 Department of Cardiology, Thoraxcentrum Twente, MST, Koningsplein 1, 7512KZ Enschede, The Netherlands; 2 Department Health Technology and Services Research, MIRA, Institute for Biomedical Technology and Technical Medicine, University of Twente, Enschede, The Netherlands; 3 Medlon Laboratory Diagnostics, Medisch Spectrum Twente, Enschede, The Netherlands; 4 Department of Cardiology, Academisch Medisch Centrum, Amsterdam, The Netherlands

**Keywords:** Endothelial function, Reactive hyperemia peripheral artery tonometry, ST-segment elevation myocardial infarction (STEMI), Primary PCI, Coronary artery disease

## Abstract

Knowledge about the changes in endothelial function after ST-elevation myocardial infarction (STEMI) is of substantial interest, but serial data are scarce. The aim of the present study was to noninvasively evaluate whether endothelial function, as assessed shortly after primary percutaneous coronary intervention (PPCI) for STEMI, may improve until 12-month follow-up. This prospective observational cohort study was performed in patients in the RESPONSE randomized trial who participated in a substudy and underwent noninvasive assessment of endothelial function at 1 (baseline), 6, and 12-month follow-up after treatment of a STEMI by PPCI. The reactive hyperemia peripheral artery tonometry (RH-PAT) method was used to assess endothelial function (higher RH-PAT index signifies better function). Of the 70 study participants, who were 57.4 ± 9.7 years of age, 55 (78.6%) were male and 9 (13%) had diabetes. The endothelial function deteriorated significantly during follow-up: the RH-PAT index at baseline, 6, and 12-month follow-up was 1.90 ± 0.58, 1.81 ± 0.57, and 1.69 ± 0.49, respectively (*p* = 0.04). Although patients were carefully treated in outpatient clinics and adequate pharmacological therapy was prescribed, we noted an increase in total cholesterol (*p* = 0.001), LDL cholesterol (*p* = 0.002), HbA1C (*p* = 0.054), and diastolic blood pressure (*p* = 0.047) However, multivariate analysis revealed that this increase in cardiovascular risk factors could not explain the observed deterioration in endothelial function. In patients with STEMI, we observed a significant deterioration in endothelial function during 12 months after PPCI that could not be explained by changes in the traditional cardiovascular risk profile.

## Introduction

Coronary heart disease remains the leading cause of morbidity and mortality in societies with a Western lifestyle [1]. Patients who experienced an ST-segment elevation myocardial infarction (STEMI) and were treated by primary percutaneous coronary intervention (PPCI) still bear a substantially increased risk of another cardiovascular event, despite all efforts to adequately treat them and to modify their cardiovascular risk factors [2]. Endothelial dysfunction, which develops in conduction and resistance vessels of patients with atherosclerosis, has been associated with an increased cardiovascular risk [3–5]. In patients with a recent STEMI, the assessment of endothelial function and its changes over time might help identify patients who require a further optimization of risk factors or an individualized therapy. Nevertheless, so far, data of changes in endothelial function in patients with a recent STEMI are extremely rare. An important first step could be to obtain such serial data in a population of STEMI patients and to evaluate the relation between changes in endothelial function and traditional cardiovascular risk factors.

Several techniques are available for a noninvasive assessment of peripheral endothelial function, which has been shown to correlate well with coronary endothelial function [6, 7]. While the ultrasound-based assessment of flow-mediated dilatation of the brachial artery requires significant training and experience, reactive hyperemia peripheral artery tonometry (RH-PAT) is an operator-independent method that has proved its value in comparison with an ultrasound-based approach and with acetylcholine-based assessment of coronary endothelial function [8, 9]. An RH-PAT index of peripheral endothelial function was shown to be reduced in the presence of proven dysfunction of the coronary endothelium [9].

In the present prospective substudy of the RESPONSE (Randomised Evaluation of Secondary Prevention by Outpatient Nurse SpEcialists) trial [10], we obtained serial data on endothelial function and evaluated the hypothesis that, following a STEMI, endothelial dysfunction may improve under medical therapy (e.g., with statins or angiotensin converting enzyme inhibitors) and standard measures of secondary prevention [11]. For that purpose, we used the RH-PAT approach in patients with a recent STEMI and assessed endothelial function at 1-, 6- and 12-month follow-up after PPCI.

## Methods

### Study population and design

This prospective cohort study was performed in 70 STEMI patients in the RESPONSE trial [10], who underwent treatment by PPCI for acute STEMI (≤ 12 h after symptom onset) and noninvasive assessment of endothelial function with the RH-PAT method at 1-, 6- and 12-month follow-up. PPCIs were performed at Thoraxcentrum Twente, the Netherlands. Of a total of 75 STEMI patients in the RESPONSE trial with RH-PAT measurements, 70 had analyzable serial RH-PAT registrations.

Patients were eligible for enrollment if they: were between 18 and 80 years of age; were available for and willing to adhere to the follow-up procedures; had no surgery or additional PCI planned within 8 weeks from PPI; and had a life expectancy of at least 2 years in the absence of heart failure NYHA class III or IV. Details of the randomized RESPONSE trial have previously been reported [10].

As inflammation and repair processes of the infarcted myocardium might have disturbed endothelial function measurements during the first weeks after the STEMI and endothelial dysfunction has not been fully recovered under medication, endothelial function was first assessed during 1-month follow-up (i.e., 4–6 weeks after PPCI) [12–14]. All patients were seen in the outpatient clinic, where noninvasive assessment of the endothelial function was performed according to strict rules in a dedicated laboratory [15]. All patients provided written informed consent for participation in the substudy. The study complied with the Declaration of Helsinki for investigation in human beings and was approved by the Medical Ethical Committee Twente in Enschede, the Netherlands.

### Coronary intervention and concomitant medical therapy

Patients were treated in the ambulance with an intravenous bolus of 5.000 IE of unfractionated heparin, a loading dose of ≥ 300 mg of acetylsalicylic acid (orally or intravenously) and an oral loading dose of 600 mg of clopidogrel. In 50 patients, a weight-adjusted intracoronary bolus of abciximab was administered after visualizing the culprit coronary artery. PPCI procedures were generally performed via the femoral route through 6F sheaths. Pharmacological therapy, use of aspiration catheters, lesion preparation (vs. direct stenting), and stent postdilatation were performed according to current guidelines and the operator’s judgment and discretion. Following primary PCI, a heart team carefully assessed the coronary angiographies and, if required, patients underwent a staged PCI for additional coronary lesions, which was generally performed within 1–2 weeks from primary PCI.

### Noninvasive assessment of endothelial function with the RH-PAT method

Endothelial function was evaluated with the RH-PAT method. The finger pulse wave amplitude was assessed with the EndoPAT-2000 sensing device and finger plethysmographic probes (Itamar Medical, Caesarea, Israel), both at baseline and during ischemia-induced hyperemia. All measurements were performed in the early morning in a dedicated laboratory after patients had fasted for at least 8 h. Patients also had to refrain from caffeine consumption, smoking, and vasoactive medications. At least 15 min prior to testing, blood pressure was measured and a blood sample was drawn in the control arm. Before any measurement, patients had an acclimatization period of 20 min in a quiet room, lying in a hospital bed at an ambient temperature of 21–23 °C.

The RH-PAT method has previously been reported in detail [15, 16]. In brief, measurements were performed using probes on the index fingers of both the study and control arms. Baseline measurements were recorded for 5 min prior to inducing ischemia by inflating a blood pressure cuff on the upper arm of the study arm for 5 min to supra-systolic pressures. After the transient arterial occlusion, the increase in blood flow in the finger of the study arm was assessed, serving as an index of the endothelium-dependent vasodilator function. The ratio of the pulse amplitude of the hyperemic finger and the baseline amplitude was calculated. Subsequently, that ratio was divided by the corresponding ratio, obtained in the control arm, to calculate the RH-PAT index (high values indicate good endothelial function). The maximum hyperemic response can be expected 90–120 s after cuff deflation [17]. Therefore, in the present study, the reactive RH-PAT index was calculated as the ratio of the mean hyperemic pulsed wave analysis over a period of 30 s, beginning at 90 s after cuff deflation, divided by the baseline pulsed wave analysis (mean baseline measurements for 3.5 min), and normalized to the concurrent measurements of the control arm [17].

### Characteristics of the subjects and the definitions used

The following information was documented at baseline: RH-PAT; age; sex; body mass index (BMI, kg/m^2^); arterial hypertension (blood pressure of > 140/90 mmHg or treatment with anti-hypertensive medication); history of smoking (previous or current smoker); history of previous myocardial infarction and/or coronary revascularization by means of PCI or bypass surgery; the presence of diabetes mellitus (patient history and/or treatment with insulin or oral anti-glycemic agents); and history of hypercholesterolemia or treatment with lipid-lowering drugs. Procedure details were also documented. At follow-up, RH-PAT and systolic blood pressure, diastolic blood pressure, and BMI were measured; current smoking habit and medication use were documented. Blood samples were analyzed for the measurements of total cholesterol, HDL cholesterol, LDL cholesterol, triglycerides, fasting glucose, and HbA1c. Patients were instructed to fast for a period of 8 h prior to drawing of the blood sample. At the three times of follow-up, we assessed the proportion of patients on adequate cardiovascular risk factor control, according to the 2003 European guidelines of cardiovascular disease prevention [11].

### Statistical analyses

Data are presented as frequencies (%) or mean ± standard deviation (SD). Between-group comparisons of RH-PAT were conducted using longitudinal mixed models. Time was treated as a categorical variable (baseline, 6, and 12 months). Compound symmetry covariance structure was used for time. Only those variables that significantly modified over time were included in the subsequent multivariate analyses. As repeated covariates, we included Hb1Ac, diastolic blood pressure, total cholesterol, and LDL cholesterol. A *p* value < 0.05 was considered statistically significant. All analyses were conducted using SPSS version 23.0 (SPSS Inc., Chicago, IL, USA).

## Results

### Characteristics of study population and treatment

Patient characteristics at baseline are displayed in Table 1. The study population had a mean age of 57.4 ± 9.7 years, and most patients were men (78.6%). Patients had a BMI of 28.0 ± 4.0 kg/m^2^ at inclusion; 40.0% were smokers while 12.9% had diabetes, 28.6% hypertension, and 24.3% hypercholesterolemia. Eight patients (11.4%) had a history of previous PCI. The index PPCI procedure was performed through the femoral route in 98.4% of the patients with an ischemia time of 155.9 ± 105.5 min. Of all patients, 73.9% had single-vessel disease and 89.9% had a left ventricular ejection fraction > 50% prior to discharge (Table 1). At discharge after PPCI, European guideline-suggested medication was prescribed as follows: 95.5% of all patients were prescribed acetylsalicylacid, 95.7% clopidogrel, 85.1% beta-blockers, 49.3% angiotensin converting enzyme inhibitors, 13.4% angiotensin receptor blockers, and 94.0% statins (Table 2).

**Table 1:** Characteristics of the study population

Patients	70 (100)
Male sex	55 (78.6)
Age (years, mean ± SD, min–max)	57.4 ± 9.7, 38.3–78.8
Body mass index (kg/m^2^, mean ± SD, min–max)^b^	28.0 ± 4.0, 21.0–42.4
Current smoker	28 (40.0)
Diabetes mellitus	9 (12.9)
Hypertension	20 (28.6)
Hypercholesterolemia	17 (24.3)
History of percutaneous coronary intervention	8 (11.4)
History of coronary artery bypass grafting	1 (1.4)
Maximum level of serum creatinine kinase^a^	
< 500 U/l	24 (34.3)
500–1,000 U/l	14 (20.0)
1,000–2,000 U/l	13 (18.6)
> 2,000 U/l	17 (24.3)
Left ventricular ejection fraction^a^	
> 50%	62 (89.9)
40–50%	7 (10.1)
< 40%	0 (0)
NYHA class^a^	
I	66 (95.7)
II	3 (4.3)
III	0
IV	0
Vascular access via femoral route^c^	63 (98.4)
Ischemia time (minutes, mean ± SD, min–max)^c^	155.9 ± 105.5, 20–600
Systolic blood pressure (mmHg, mean ± SD, min–max)^c^	124.6 ± 24.5, 60–170
Diastolic blood pressure (mmHg, mean ± SD, min–max)^c^	76.6 ± 14.7, 35–110
Heart rate during (beats/min, mean ± SD, min–max)^c^	73.8 ± 18.0, 46–115
Extent of coronary artery disease^a^	
1 Vessel disease	51 (73.9)
2 Vessel disease	15 (21.7)
3 Vessel disease	3 (4.3)
Type of stents implanted^c^	
Bare metal stents	42 (67.7)
Drug-eluting stents	18 (29.0)
Bare metal and drug-eluting stents	2 (3.2)
Total stent length (mm, mean ± SD, min–max)^c^	34.4 ± 23.9, 13–132

Data are number (%) unless otherwise stated. Baseline characteristics are reported

*NYHA* New York Heart Association class of heart failure, *STEMI* ST-segment elevation myocardial infarction

^a^1 missing

^b^2 missings

^c^4–10 missings

**Table 2:** Serial data of endothelial function, variable patient characteristics, and medication prescribed

Variables	Baseline	6 months	12 months	*P* value
Endothelial function RH-PAT index, mean ± SD	1.90 ± 0.58	1.81 ± 0.57	1.69 ± 0.49	0.04*
Variable patient characteristics				
Body mass index > 25 kg/m^2^	52 (76.5)	52 (80.0)	53 (79.1)	0.71
Current smoker	29 (41.4)	23 (33.8)	25 (36.8)	0.92
Systolic blood pressure > 140 mmHg	15 (21.7)	23 (33.8)	20 (29.0)	0.34
Diastolic blood pressure > 90 mmHg	15 (21.7)	13 (19.1)	19 (27.5)	0.48
Total cholesterol > 4.5 mmol/l	7 (10.0)	8 (11.4)	8 (11.6)	0.76
LDL cholesterol > 2.5 mmol/l	10 (14.3)	8 (11.4)	8 (11.6)	0.63
HDL cholesterol < 1.0 mmol/l	32 (45.7)	29 (41.4)	25 (36.2)	0.26
Triglycerides > 2.0 mmol/l	5 (7.1)	6 (8.6)	10 (14.5)	0.15
Medication prescribed				
Acetylsalicylic acid	64 (95.5)	63 (94.0)	63 (94.0)	0.70
Clopidogrel	67 (95.7)	62 (93.9)	39 (57.4)	< 0.001
Oral anticoagulant	3 (4.5)	2 (3.0)	2 (3.0)	0.64
Beta blocker	57 (85.1)	48 (71.6)	48 (71.6)	0.07
ACE inhibitor	33 (49.3)	36 (57.1)	34 (50.7)	0.86
ARB	9 (13.4)	12 (18.8)	9 (13.4)	1.00
Calcium antagonist	10 (14.5)	13 (19.7)	9 (13.2)	0.84
Statin	63 (94.0)	63 (98.4)	63 (94.0)	1.00

Data are number (%) unless otherwise stated. Baseline assessment was performed 4–6 weeks after the acute ST-elevation myocardial infarction

*ACE* angiotensin converting enzyme, *ARB* angiotensin receptor blocker, *HDL* high density lipoprotein, *LDL* low density lipoprotein

*P value obtained from longitudinal mixed model analysis

### Endothelial function in serial measurements

The endothelial function decreased over time, starting at a mean RH-PAT index of 1.90 ± 0.58 at baseline, to 1.81 ± 0.57 at 6-month follow-up, and eventually to 1.69 ± 0.49 at 12-month follow-up (*p* = 0.04 in mixed model). Further details of the RH-PAT index measurements are displayed in Table 1.

### Relation between changes in risk factors and endothelial function

Table 3 indicates, for several variables, the proportion of patients who were not on adequate risk factor control, according to the 2003 European guidelines of cardiovascular disease prevention. Despite comprehensive medical treatment, the number of patients did not change over time. From 6 to 12 months, there was only a significant decrease in the use of clopidogrel (*p* < 0.01).

**Table 3:** Differences in endothelial function and risk factors over time compared to baseline values

Variables	Baseline	∆ at 6 months	∆ at 12 months	*P* value*
Endothelial function RH-PAT index	1.90 (1.77; 2.03)	− 0.09 (− 0.25; 0.07)	− 0.21 (− 0.36;− 0.04)	0.04
Body mass index (kg/m^2^)	28.0 (27.1; 29.0)	0.12 (− 0.33;0.58)	0.16 (− 0.34;0.67)	0.76
HbA1c (mmol/l)	6.01 (5.80; 6.21)	0.02 (− 0.07; 0.11)	0.13 (0.00; 0.26)	0.054
Systolic BP (mmHg)	131.2 (127.5; 135.0)	2.1 (− 2.6; 6.7)	1.9 (− 2.8; 6.5)	0.54
Diastolic BP (mmHg)	79.6 (76.9; 82.4)	2.3 (− 1.0; 5.7)	3.7 (0.3; 7.1)	0.047
Total Cholesterol (mmol/l)	3.41 (3.22; 3.59)	0.31 (0.11; 0.52)	0.27 (0.11; 0.44)	< 0.001
LDL Cholesterol (mmol/l)	1.71 (1.57; 1.85)	0.25 (0.08; 0.42)	0.17 (0.04; 0.30)	0.002
HDL Cholesterol (mmol/l)	1.06 (1.01; 1.13)	0.03 (− 0.02; 0.07)	0.02 (− 0.03; 0.07)	0.45
Triglycerides (mmol/l)	1.39 (0.21; 1.56)	0.07 (− 0.12; 0.27)	0.17 (− 0.05; 0.40)	0.19

Data are mean (95% confidence interval)

*∆* difference from baseline, *BP* blood pressure, *HbA1c* glycated hemoglobin, *HDL* high density lipoprotein, *LDL* low density lipoprotein

*P values obtained from longitudinal mixed model analysis

A longitudinal mixed model analysis shows that, of all cardiovascular risk factors assessed, the following worsened significantly over time: HbA1c; diastolic blood pressure; total cholesterol; and LDL cholesterol (Table 4). However, multivariate analysis demonstrated that none of the cardiovascular risk factors was found to be responsible for the decrease in endothelial function over time. Results of the multivariate analysis are displayed in Table 4.

**Table 4:** Prognostic factors of changes in endothelial dysfunction

Variables	Est (SE)	*P* value
Intercept	1.85 (0.46)	
Time baseline (reference)		
Time 6 months	− 0.09 (0.09)	0.28
Time 12 months	− 0.14 (0.10)	0.14
HbA1c	− 0.018 (0.052)	0.73
Diastolic blood pressure	− 0.002 (0.004)	0.64
Total cholesterol	0.114 (0.107)	0.29
LDL cholesterol	− 0.054 (0.133)	0.68
Clopidogrel	− 0.15 (0.12)	0.21

*Est* (*SE*) estimated (standard error), *HbA1c* glycated hemoglobin, *LDL* low density lipoprotein

## Discussion

### Main findings

Following STEMI treated by PPCI, endothelial function—as measured by RH-PAT—did not improve from baseline at 1–12-month follow-up, despite the prescription of guideline-recommended medical therapy [11] in the majority of patients and counseling for cardiovascular risk factor reduction. In the present study population, endothelial function even decreased over time. Further statistical analyses found no relation between this deterioration in endothelial function and changes in traditional cardiovascular risk factors, of which some also showed deterioration over time. The findings of the present study are unique, as it is the first to report long-term serial endothelial function measurements *in STEMI patients* during a period of 12 months after PPCI.

### Approaches to serially assess endothelial function

Through the years, several methods have been developed to identify subjects with disturbed endothelial function, as they are at an increased risk of cerebrovascular [18] and cardiovascular events [19, 20]. Coronary angiographic assessment after intracoronary injection of acetylcholine remains the “gold standard”. The main limitations of this catheter-based approach are its invasiveness and inherent risk of complications. Moreover, it is not readily accessible in daily clinical practice. However, peripheral endothelial function correlates well with coronary endothelial function, reflecting the systemic nature of atherosclerosis [6, 21]. Therefore, noninvasive methods were developed to assess endothelial health, all of which apply the principle of reactive hyperemia in response to ischemia in one of the upper extremities. The reactive hyperemia response of the brachial artery can be measured using high-frequency ultrasound, an approach that has proven to be accurate and reproducible in experienced hands [22]. This has previously been shown to predict the likelihood of coronary disease in patients with chest pain and estimate the long-term risk of cardiovascular events [19, 23].

Another method of measuring the reactive hyperemic response to ischemia is RH-PAT. Data obtained with RH-PAT were shown to correlate well with both findings of the high-frequency ultrasound method and coronary endothelial function [9, 10, 20]. After the identification of patients with endothelial dysfunction, who are known to be at an increased risk of developing cardiovascular events [4], aggressive modification of traditional cardiovascular risk factors might improve patient outcome [24, 25]. Such improvement in outcome has been attributed to functional improvement of the endothelium, while reversal of atherosclerosis-related structural changes to the vessel wall is more difficult to achieve and may, to a much lesser extent, account for an improvement in clinical prognosis [26].

### Previous studies with serial endothelial function measurement

Other research groups have performed serial endothelial function measurements in different study populations, assessing changes during a period of 3–6 months. Kitta et al. [27] previously examined 251 patients with newly diagnosed stable coronary disease and reduced flow-mediated dilatation of the brachial artery, showing that, in four out of ten patients, impairment of endothelial function persisted at 6-month follow-up despite optimal medical therapy. Persistent impairment of endothelial function and the presence of diabetes had an independent adverse effect on clinical outcome after 31 ± 4 months. However, that study did not include patients with recent STEMI or other acute coronary syndromes. Nevertheless, similar to the observations of our present study, the authors found no relation between alterations in traditional risk factors in response to medical therapy and changes in endothelial function during 6 months [27].

The phenomenon of persistent impairment of endothelial function was also seen in 245 patients with chronic heart failure, in whom endothelial function was serially assessed during 6 months by flow-mediated arterial vasodilatation [28]. Despite optimal medical therapy, persistent impairment of endothelial function was seen in 53% of patients, while 47% showed an improvement. Again, there was no significant difference in clinical parameters or traditional cardiovascular risk factors between patients with persistently impaired versus improved endothelial function [28]. On the contrary, Careri et al. found that endothelial function of 60 patients with non-ST-elevation myocardial infarction (by assessment of flow-mediated arterial dilation) improved during no more than 3 months to a level that was similar to that of 40 subjects with stable coronary disease [29].

Our present study (in STEMI patients) as well as most of the previous studies (in other patient populations than STEMI patients) suggest that the continuum of endothelial dysfunction can ultimately reach a stage from which—despite adequate or even optimal medical therapy and risk factor control—restoration is unlikely or even impossible. At that point, the total load of atheroma, genetic predisposition, or unknown other risk factors may become increasingly important for the further course of endothelial function. While we found endothelial function to decrease over time, the majority of patients did not fulfill the RH-PAT method-based criterium of a significant endothelial dysfunction (i.e., RH-PAT index < 1.67) [9]. In addition, we can only speculate that deterioration in medication compliance or the recommencement of smoking also could have played a role.

### Study limitations

Use of the femoral access route in 98% of our patients may be considered an advantage [30]. Nevertheless, our study is limited by the size of the study population. In addition, in patients with STEMI, the optimal timing of endothelial function measurement might be a matter of debate. We performed the baseline study at 1-month follow-up in order to ensure that inflammation and repair processes of the infarcted myocardium [12–14] could not disturb our measurements. Yet, we acknowledge that we cannot rule out that endothelial function may have improved during the period from the PPCI to the first RH-PAT measurement, for instance due to favorable effects of the cessation of smoking or the prescribed drugs, such as statins which may improve endothelial function in patients with coronary artery disease [31] and reduce cardiovascular risk in STEMI patients [32]. For that reason, the slight but statistically significant deterioration of endothelial function from baseline to 12-month follow-up could theoretically represent a relapse of the endothelial function to levels that preexisted just before the STEMI. In addition, the achieved levels of lipid lowering reflect recommendations of the period during which the study participants were treated, while current guidelines recommend an even more aggressive treatment that might improve endothelial function. Finally, we examined the impact of traditional cardiovascular risk factors on endothelial function, but we did not evaluate alcohol intake, vitamin D levels, or the levels of residual chest pain, which also might have been of interest [33–35].

## Conclusions

In patients with STEMI, we observed during 12 months after PPCI a significant deterioration in endothelial function, as measured by the RH-PAT method, which could not be explained by changes in traditional cardiovascular risk profiles. These findings suggest that the continuum of endothelial dysfunction can ultimately reach a stage from which restoration is unlikely.
